# Activation of a bacterial killing machine

**DOI:** 10.1371/journal.pgen.1009261

**Published:** 2021-01-07

**Authors:** Felicity Alcock, Tracy Palmer

**Affiliations:** Microbes in Health and Disease Theme, Biosciences Institute, Newcastle University, Newcastle upon Tyne, United Kingdom; Swiss Federal Institute of Technology Lausanne (EPFL), SWITZERLAND

Protein secretion systems are multi-subunit machines located in the cell envelopes of prokaryotes. They provide a fundamental route through which bacteria interact with their surroundings. Outside of the laboratory, bacteria are rarely found in monoculture, usually existing in mixed-species communities. Some of the most complex bacterial communities are found in association with higher eukaryotes; for example the human gastrointestinal tract represents one of the most densely colonised habitats described to date (e.g., [1]). Within such a community, bacteria interact not only with other microorganisms but often also with host components. Many of these interactions are mediated through secreted proteins.

**Fig 1:**
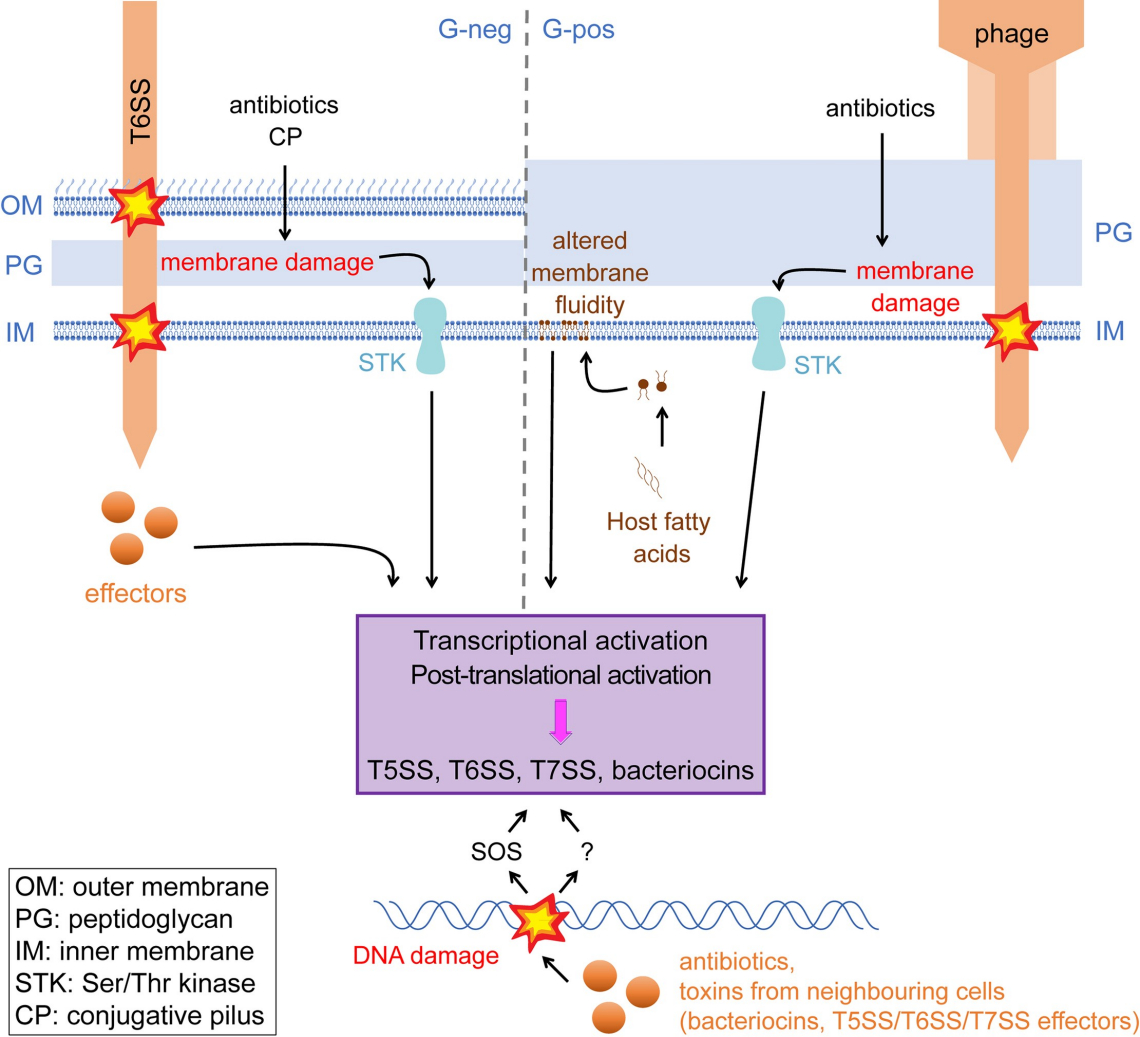
Pathways for activation of interbacterial antagonism. A bacterial cell’s first line of defence against attack from competitors is the cell envelope. Breach of this barrier by an incoming T6SS, conjugative pilus or phage, or antibiotics which target the cell membranes activates specific responses in gram-negative and gram-positive bacteria. Such signals are integrated by the cell, in some cases via a membrane Ser/Thr kinase, to mediate transcriptional and posttranslational activation of retaliatory type VI and type VII secretion. An alternative trigger for T7SS activation is decreased membrane fluidity resulting from the incorporation of host fatty acids. Intracellular translocation of competitor effectors can also trigger antagonism. DNA damage mediated by competitor-produced nucleases or DNA-targeting antibiotics can also stimulate T6SS and T7SS transcription and bacteriocin production. The latter is known to be activated via the SOS response. CP, conjugative pilus; G-neg, gram-negative; G-pos, gram-positive; IM, inner membrane; OM, outer membrane; PG, peptidoglycan; STK, Ser/Thr kinase; T5SS, type V protein secretion system; T6SS, type VI protein secretion system; T7SS, type VII protein secretion system.

Interbacterial competition plays a key role in the dynamics of microbial communities and bacterial colonisation. Toxins targeting a variety of cellular functions are secreted from aggressor cells either via specific secretion machineries or, in the case of colicins and pyocins, through lysis of the producing cell. In both cases, the costs of toxin production and secretion are high, and expression of the requisite genes are tightly regulated [2,3]. In gram-negative bacteria, type V and type VI secretion systems (T5SS and T6SS) mediate interbacterial antagonism via toxin delivery to neighbouring cells. The antibacterial activity of the T6SS promotes colonisation by the producing organism (e.g., [4]). Regulation of the T6SS can be pre- or posttranslational depending on the system. In *Pseudomonas aeruginosa* assembly of the T6SS is dynamic and controlled through cycles of threonine phosphorylation mediated via a membrane-bound kinase termed PpkA (reviewed in [5]). The activity of PpkA is modulated by cell envelope damage—specifically, it is activated by incoming T6SS attack from a neighbouring bacterium, thereby promoting T6-dependent counterattack [6]. Membrane damage from an incoming conjugative pilus or polymyxin B can likewise trigger this response [7]. A specific toxin effector from a competitor T6SS has also been shown to activate the T6SS [8] in a manner reminiscent of colicin activation by the SOS response following colicin-mediated DNA damage (Fig 1). Additional signals from quorum sensing and nutrient availability are also integrated into these control mechanisms [9].

Many gram-positive bacteria produce a type VII protein secretion system (T7SS). Studies in mycobacteria have shown that the system plays a critical role in pathogenesis and intracellular trafficking [10]. However, there is a growing appreciation that firmicutes utilise the T7SS primarily for bacterial antagonism. Two T7-dependent antibacterial toxins have been identified and characterised in *Staphylococcus aureus* and three in *Streptococcus intermedius* [11–13]. In most firmicutes studied to date, the T7SS appears to be carefully regulated and is poorly active under laboratory conditions [14,15]. For example, the *S*. *aureus* T7SS locus is under complex transcriptional control [16] and is up-regulated in response to decreased membrane fluidity [17]. It is also regulated at the posttranslational level as secretion activity is generally very inefficient, but can be stimulated in some strain backgrounds by the presence of hemin [18].

The study by Chaterjee and colleagues has investigated environmental cues that activate the T7SS in the firmicute *Enterococcus faecalis* [19], a bacterium found in the gut microbiota of healthy individuals, which is an opportunistic pathogen capable of causing severe infection [20]. Previous work investigating phage therapy as an alternative treatment for drug-resistant *E*. *faecalis* noted that phage-infected cells induce expression of the T7SS locus [21]. Here the authors investigated whether phage infection could act as a trigger for T7SS-dependent attack of neighbouring bacteria. They showed that indeed incubation of *E*. *faecalis* with phage resulted in a 1,000-fold reduction in recovery of a phage-resistant bystander strain, which was absolutely dependent on a functional T7SS in the attacker strain. Phage-induced antibacterial antagonism was shown to require contact between attacker and target strains and to be mediated by at least one toxin from the LXG family of T7SS substrates.

To investigate the mechanism of phage-induced T7SS activation, the authors determined whether antibiotics targeting the cell envelope could also induce T7SS gene expression. While cell wall–targeting antibiotics had little effect on T7SS expression, daptomycin which targets the cell membrane resulted in T7SS gene up-regulation. Curiously, a similar up-regulation was also seen when cells were treated with DNA-targeting antibiotics ciprofloxacin or mitomycin C. In agreement with the observed up-regulation of the T7SS genes, treatment with daptomycin was also shown to induce T7SS-dependent inhibition of bystander bacteria ([19]; Fig 1).

Since both phage infection and daptomycin treatment cause membrane damage, the authors sought to identify how this signal was transduced to activate gene expression. The membrane-bound serine–threonine kinase, IreK, is well known to play a role in *E*. *faecalis* cell envelope homeostasis [22]. In a Δ*ireK* strain, the T7SS genes were no longer induced upon phage predation, and phage-induced antibacterial antagonism was abolished [19].

Collectively, these results demonstrate that *E*. *faecalis* has a potent antibacterial T7SS that is transcriptionally activated through a membrane-bound kinase by environmental conditions that trigger membrane damage. This has intriguing parallels with the activation of the antibacterial T6SS in gram-negative bacteria, where activity is also controlled through a membrane-bound kinase that senses damage associated with incoming T6-dependent attack (Fig 1). However, in the case of *Enterococcus*, activation is at the level of gene expression rather than the posttranslational control of secretion activity seen for the T6SS. It is tempting to speculate that T7 attack may also lead to the up-regulation of the *E*. *faecalis* T7SS. In this respect, it is interesting that DNA damaging agents also induce T7 gene expression—DNA is a common target for antibacterial toxins (e.g., [11]), and it has been reported that a DNA-targeting toxin of the contact-dependent T5SS can modulate gene expression in neighbouring cells, including a locus encoding a predicted T6SS [23]. Investigating the link between DNA damage and T7 gene expression would be an exciting avenue for future research.

Among the observations made by Chatterjee and colleauges, it was surprising to note that cell wall targeting antibiotics did not affect regulation of the T7 genes [19]. This is perhaps unexpected since it is well documented that IreK responds to cell wall stresses [24]. This indicates that IreK has distinct input/output pathways, as suggested by other studies (e.g., [25]). The network of interactions integrated through IreK and the precise mechanism by which it controls expression of the T7 locus certainly warrant further investigation.

Regulated control of T7 secretion activity appears to be a common thread across almost all of the firmicute T7SSs investigated to date. Both the present study and prior work in *S*. *aureus* have linked changes at the cell membrane to the regulation of secretion system expression. It will be interesting to determine whether other T7SSs are controlled in a similar manner and how posttranslational control of secretion is achieved.

Phage therapy is considered as a potential treatment for multidrug-resistant pathogens, and their narrow host range has led to the belief that they could be utilised for the selective removal of individual pathogens with little effect on the microbiota [26]. The work described here has shown that phage treatment may have unexpected consequences for other members of microbial communities.
